# Genetic Dissection of Budding Yeast PCNA Mutations Responsible for the Regulated Recruitment of Srs2 Helicase

**DOI:** 10.1128/mbio.00315-23

**Published:** 2023-03-02

**Authors:** Li Fan, Wenqing Zhang, Josephine Rybchuk, Yu Luo, Wei Xiao

**Affiliations:** a Beijing Key Laboratory of DNA Damage Responses and College of Life Sciences, Capital Normal University, Beijing, China; b Department of Biochemistry, Microbiology and Immunology, University of Saskatchewan, Saskatoon, Saskatchewan, Canada; c Toxicology Program, University of Saskatchewan, Saskatoon, Saskatchewan, Canada; Harvard Medical School

**Keywords:** *Saccharomyces cerevisiae*, PCNA, DNA-damage tolerance, Rad5, Rad18, Srs2, PIP box, budding yeast

## Abstract

DNA-damage tolerance (DDT) is a mechanism by which eukaryotes bypass replication-blocking lesions to resume DNA synthesis and maintain cell viability. In Saccharomyces cerevisiae, DDT is mediated by sequential ubiquitination and sumoylation of proliferating cell nuclear antigen (PCNA, encoded by *POL30*) at the K164 residue. Deletion of *RAD5* or *RAD18*, encoding two ubiquitin ligases required for PCNA ubiquitination, results in severe DNA-damage sensitivity, which can be rescued by inactivation of *SRS2* encoding a DNA helicase that inhibits undesired homologous recombination. In this study, we isolated DNA-damage resistant mutants from *rad5*Δ cells and found that one of them contained a *pol30-A171D* mutation, which could rescue both *rad5*Δ and *rad18*Δ DNA-damage sensitivity in a *srs*2-dependent and PCNA sumoylation-independent manner. Pol30-A171D abolished physical interaction with Srs2 but not another PCNA-interacting protein Rad30; however, Pol30-A171 is not located in the PCNA-Srs2 interface. The PCNA-Srs2 structure was analyzed to design and create mutations in the complex interface, one of which, *pol30-I128A*, resulted in phenotypes reminiscent of *pol30-A171D*. This study allows us to conclude that, unlike other PCNA-binding proteins, Srs2 interacts with PCNA through a partially conserved motif, and the interaction can be strengthened by PCNA sumoylation, which turns Srs2 recruitment into a regulated process.

## INTRODUCTION

Living organisms are constantly exposed to spontaneous and environmental DNA damage. In addition to various DNA repair pathways, cells have also evolved to survive in the presence of replication-blocking lesions, a process known as DNA-damage tolerance (DDT). In a model lower eukaryote Saccharomyces cerevisiae, DDT is achieved by sequential ubiquitination of proliferating cell nuclear antigen (PCNA) (1, 2). In response to DNA damage, an E2-E3 complex Rad6-Rad18 monoubiquitinates PCNA at its K164 residue, which increases affinity for Y-family DNA polymerases, including Polη (3) and Rev1 (4), and promotes translesion DNA synthesis (TLS). Monoubiquitinated PCNA can be further polyubiquitinated through K63 linkage by another E2-E3 complex Mms2-Ubc13-Rad5 that leads to error-free lesion bypass (5), probably through template switch (6, 7). The same K164 residue in PCNA can also be sumoylated by an E2-E3 complex Ubc9-Siz1 in the absence of DNA damage (5), which recruits Srs2 to inhibit undesired homologous recombination (HR) (8, 9). The *srs2* (*s*uppression of *r*ad-*s*ix) mutant was initially isolated by its ability to rescue the severe DNA-damage sensitivity of *rad6* and *rad18* mutants (10) and subsequently found to encode a DNA helicase (11) that inhibits hyper-recombination (12) by preventing Rad51-ssDNA filament formation (13, 14). Srs2 also suppresses the severe DNA-damage sensitivity of other DDT pathway mutants, including *rad5* (15–17). Since the *srs2* suppression of DNA-damage sensitivity is limited to only DDT pathway mutants and acts to channel DNA lesions to HR (18), loss of Srs2 is thought to activate a salvage HR pathway (19, 20).

PCNA forms a toroidal-shaped homotrimer that interacts with the DNA strand by encircling it, forming a sliding clamp that recruits a large number of proteins to the DNA replication fork not only for replication, but also for other processes, including cell cycle regulation, recombination and DNA damage response (DDR) (21). The most common PCNA-binding motif is known as the PCNA interaction protein (PIP) box, consisting of a consensus sequence Q-x-x-(h)-x-x-(a)-(a) (where “h” represents amino acid with moderately hydrophobic side chains like L, I, M; “a” represents amino acid with highly hydrophobic, aromatic side chains like F, Y; and “x” represents any residues) (22). Several proteins involved in the yeast DDT pathway, including Srs2 (23), Polη (encoded by *RAD30*) (24, 25), Pol2 and all three Polδ subunits (26), contain a PIP box or its variants. Correspondingly, analysis of the PCNA-PIP box complex structure (27) reveals that PCNA interdomain connecting loop (IDCL) and C-terminal regions mediate its interaction with the PIP box.

We systematically analyzed DNA-damage resistant isolates from *rad5* and *rad18* null mutants. While majority of these isolates contained mutations in the *SRS2* gene as anticipated, several isolates from *rad5Δ* cells did not contain *srs2* mutation. Whole-genome sequencing analyses identified putative mutations responsible for DNA-damage tolerance phenotype, among which a missense mutation in *POL30* encoding yeast PCNA is further investigated in this study. It was found that PCNA mutations specifically affecting its interaction with Srs2 can activate the salvage HR pathway.

## RESULTS

### Isolation and characterization of MMS-resistant colonies from *rad5Δ* and *rad18Δ* cells.

**FIG 1:**
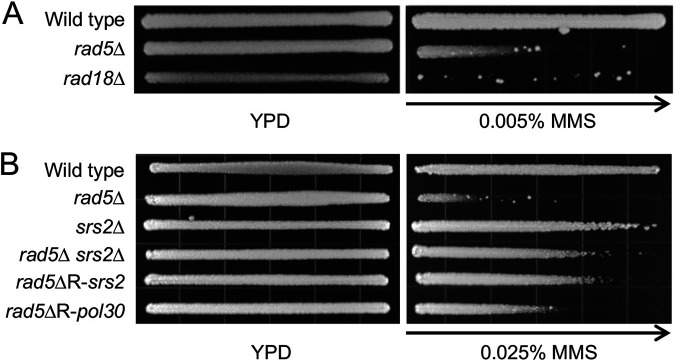
*rad5Δ*, *rad18Δ* and their MMS resistant mutant phenotypes by an MMS gradient plate assay. (A) *rad5Δ* and *rad18Δ* mutant phenotypes. (B) Relative sensitivity of MMS resistant isolates (*rad5ΔR*) from *rad5Δ* cells. Cells grown on a YPD plate serve as controls. The length of growth on the MMS-containing gradient plates measures the relative sensitivity of cells to MMS. Arrows point to increasing MMS concentration. Plates were incubated at 30°C for 2 days before photography.

Rad18 and Rad5 serve as E3s for PCNA monoubiquitination and subsequent polyubiquitination, respectively (5). In addition, Rad5 is also required for the recruitment of a TLS polymerase Rev1 (28). Hence, Rad18 and Rad5 are required for both branches of DDT, and their null mutants are extremely sensitive to DNA-damaging agents that block replication, including ultraviolet (UV) irradiation, methyl methanesulfonate (MMS), and 4-nitroquinoline N-oxide (4-NQO). It was routinely observed that in an MMS gradient plate assay, some resistant colonies appeared at the high MMS concentration end (Fig. 1A). These resistant colonies turned out to be inherited, as they became significantly more resistant than their parental cells when replating on the MMS gradient plates again (see Fig. 1B for example). It has been well known that *srs2* loss-of-function mutations can rescue *rad6*, *rad18* (10) and *rad5* (15–17) severe sensitivity. To ask if these mutants carry *srs2* mutations, we sequenced the *SRS2* and its promoter region from 33 independent MMS-resistant *rad18*Δ isolates and found that they all carried mutations in *SRS2* (data not shown). Interestingly, out of 25 independent MMS-resistant *rad5*Δ isolates (designated *rad5*ΔR), only half (13/25) carried *srs2* mutations. Genomic DNA was extracted from the 13 non-*SRS2*-mutation *rad5ΔR* isolates, and the whole-genome sequencing was performed and analyzed. Indeed, these isolates did not contain mutation in *SRS2*, and the bioinformatics analysis revealed candidate gene mutations, among which a *pol30-A171D* mutation caught our attention. Fig. 1B shows that a sample *rad5ΔR* isolate that contains a *srs2* mutation (*rad5*ΔR-*srs2*) is indistinguishable from the reconstituted *rad5Δ srs2*Δ mutant. In comparison, a *rad5*ΔR isolate containing a *pol30-A171D* (designated *rad5ΔR-pol30*) mutation is much more resistant than *rad5Δ* but less resistant than the *rad5Δ srs2*Δ mutant.

### *pol30-A171D* is synergistic to *pol30-K164R* in the rescue of *rad5Δ* and *rad18Δ* sensitivity.

To ask whether the *pol30-A171D* mutation is indeed responsible for the rescue of *rad5*Δ cells, we wished to create a *pol30-A171D rad5*Δ double mutant. However, *POL30* is an essential gene that cannot be readily deleted and replaced by a point mutation. Hence, we took a plasmid shuffling approach as described (29), in which the chromosomal *POL30* was deleted while carrying a YCp-Pol30 plasmid with a *URA3* selectable marker. Another plasmid carrying a YCp-Pol30 plasmid or its mutant derivatives with a *LEU2* selectable marker can be transformed into the above cells and the transformants lost original YCpU-Pol30 can be selected in a 5-FOA plate. The phenotype of a *pol30* mutant can then be assessed in comparison to its wild type transformant.

**FIG 2:**
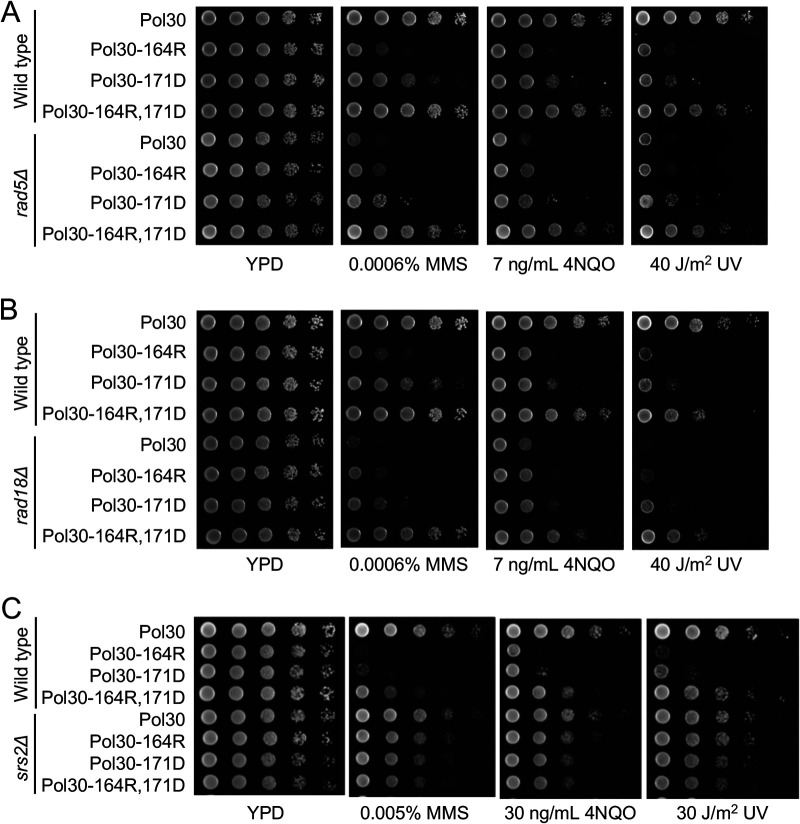
Genetic interactions between *pol30-A171D* and relevant mutations by a serial dilution assay. (A) Genetic interaction between *pol30-A171D* and *pol30-K164R* in the rescue of *rad5Δ* mutant. (B) Genetic interaction between *pol30-A171D* and *pol30-K164R* in the rescue of *rad18Δ* mutant. (C) Genetic interaction between *pol30-A171D* and *pol30-K164R* in the *srs2* background. Yeast cells cultured overnight were used to make 10-fold serial dilutions and then spotted to YPD or YPD plus various concentrations of MMS or 4NQO or exposed to UV irradiation at indicated doses, followed by incubation at 30°C for 2 days before photography. Only one representative dose for each treatment is shown.

The reconstituted *pol30-A171D* mutant displayed increased sensitivity to MMS, and it was able to rescue the severe MMS sensitivity of the *rad5*Δ mutant (Fig. 2A). The *pol30-K164R* mutation was reported to rescue the *rad18Δ* sensitivity (5). It was observed that in comparison to *pol30-K164R*, *pol30-A171D* cells were less sensitive to DNA-damaging agents both in wild-type and *rad5*Δ backgrounds (Fig. 2A). In addition, *pol30-A171D* also rescued the *rad18*Δ mutants to DNA damage (Fig. 2B).

Since Pol30-K164 is the ubiquitination and sumoylation site (5), it is believed that the *pol30-K164R* mutation rescues *rad18* sensitivity through loss of PCNA sumoylation and compromised Srs2 recruitment (8, 9). We first asked whether *pol30-A171D* affects PCNA sumoylation by a Western blot analysis, which revealed that under the same experimental conditions, *pol30-K164R* abolished sumoylation at the Pol30-K164 residue while *pol30-A171D* did not (Supplementary Fig, S1, lanes 2 and 4). To address genetic relationship between *pol30-K164R* and *pol30-A171D*, we created and tested the *pol30- K164R*,*A171D* double mutant. To our surprise, the double mutant was much more resistant to all three DNA-damaging agents than its corresponding single mutant, and fully rescued *rad5*Δ (Fig. 2A) and *rad18*Δ (Fig. 2B) mutants. In quantitative terms, the *pol30-K164R* mutation rescued *rad5Δ* or *rad18Δ* MMS sensitivity by less than 10-fold, and the *pol30-A171D* mutation rescued *rad5Δ* or *rad18Δ* MMS sensitivity by more than 10-fold, while the double mutation rescued their MMS sensitivity by as high as 10,000-fold. Hence, *pol30-K164R* and *pol30-A171D* are synergistic in rescuing *rad5*Δ and *rad18*Δ MMS, as well as 4NQO and UV sensitivity.

### The rescuing effect of *pol30-A171D* relies on *SRS2*.

The rescuing effect of *pol30-K164R* is known to rely on *SRS2*. To ask whether *pol30-A171D* also behaves the same, we tested the relative sensitivity of *pol30-A171D*, *pol30-K164R* and the corresponding double mutant in a *srs2* background. As shown in Fig. 2C, the *srs2* mutation is epistatic to all three *pol30* mutations with respect to DNA damage sensitivity. The above observations allow us to conclude that *pol30-A171D* acts in an *SRS2*-dependent manner.

### *pol30-A171D* affects Pol30-Srs2 interaction.

**FIG 3:**
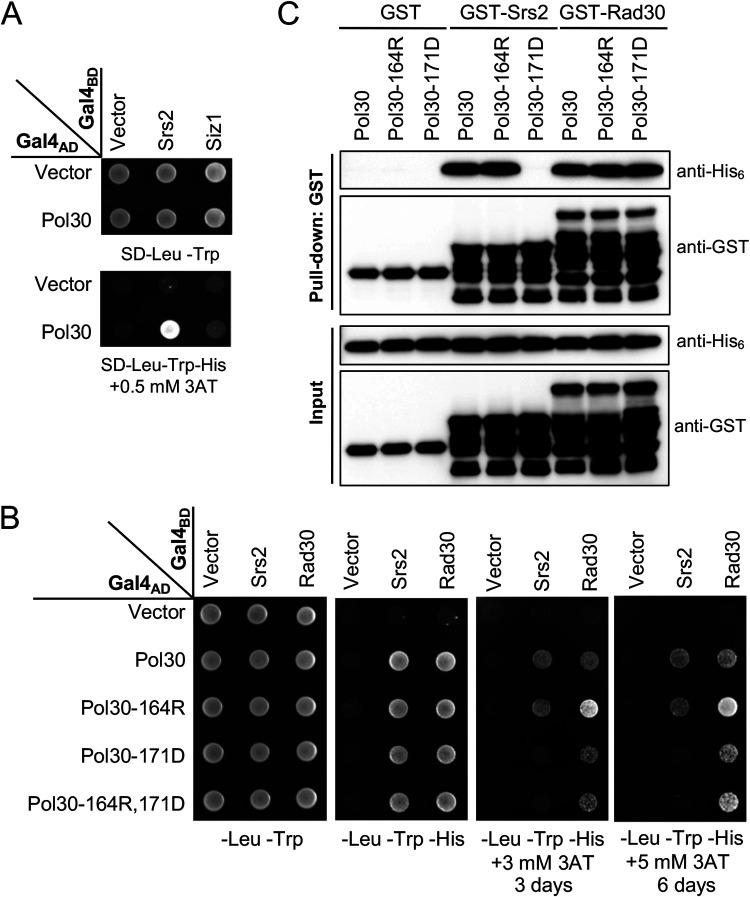
Physical interaction between Pol30-A171D and Srs2. (A) Interaction between Pol30, Srs2 and Siz1 by a Y2H assay. (B) Interaction between mutated Pol30 and Srs2, Rad30 by a Y2H assay. *SRS2*, *SIZ1* and *RAD30* ORFs were cloned in pGBT9 (Gal4_BD_), and *POL30* ORF and its mutant alleles were cloned in pGAD424 (Gal4_AD_). pGBT9 and pGAD424 derived plasmids were cotransformed into PJ69-4a, and the transformants were spotted on control (SD-Leu-Trp) and selective (SD-Leu-Trp-His with or without 3AT) plates, which were incubated at 30°C for 3 days or as indicated before photography. Independent transformants were examined in parallel on multiple selective plates and incubated for different periods. Only the representative images are shown. (C) *In vitro* interaction between Srs2 C terminus (Srs2-CT), Rad30-PIP and Pol30 or its mutant derivatives by a GST pulldown assay. Proteins before and after the GST pulldown were subjected to Western blotting by using antibodies against His_6_ and GST tags.

Through the above genetic analysis, we hypothesized that the *pol30-A171D* mutation affects Srs2 recruitment independently of PCNA sumoylation. To test this hypothesis, A yeast two-hybrid (Y2H) assay was performed to examine physical interaction between Pol30 and Srs2 as well as Siz1. Under our experimental conditions, Pol30 was found to interact with Srs2 but not Siz1 (Fig. 3A). Srs2 was then used to test its interaction with Pol30 mutations using a PIP box-containing protein Rad30 as a positive control. Fig. 3B shows that Pol30-A171D did not affect interaction with Rad30 but severely reduced interaction with Srs2. In contrast, Pol30-K164R did not affect interaction with Srs2 and even enhanced interaction with Rad30. Again, the dual substitution behaved like Pol30-A171D.

The Srs2 C-terminal domain (Srs2-CT) was reported to interact with Pol30 and SUMO through its PIP box and SIM motif, respectively (23). In a GST-pulldown assay, Srs2-CT (amino acid residues 1107 to 1174) was cloned into pGEX6 to produce GST-tagged Srs2-CT and His_6_-tagged Pol30 was produced by pET-Pol30 transformed bacterial cells. Under a robust condition of direct interaction between GST-Srs2-CT and His_6_-Pol30, Pol30-K164R did not affect this interaction while Pol30-A171D completely abolished such interaction; in contrast, neither Pol30-K164R nor Pol30-A171D affected physical interaction with the Rad30-PIP domain (Fig. 3C). In conclusion, both Y2H and GST-pulldown assay results support a notion that Pol30-A171D affects Srs2 interaction, but Pol30-K164R does not. It is noted that in both Y2H and pulldown assays, Pol30 was not sumoylated, which explains why Pol30-K164R does not affect interaction with Srs2.

### Distinct effects between *pol30-A171D* and *pol30-K127R*.

**FIG 4:**
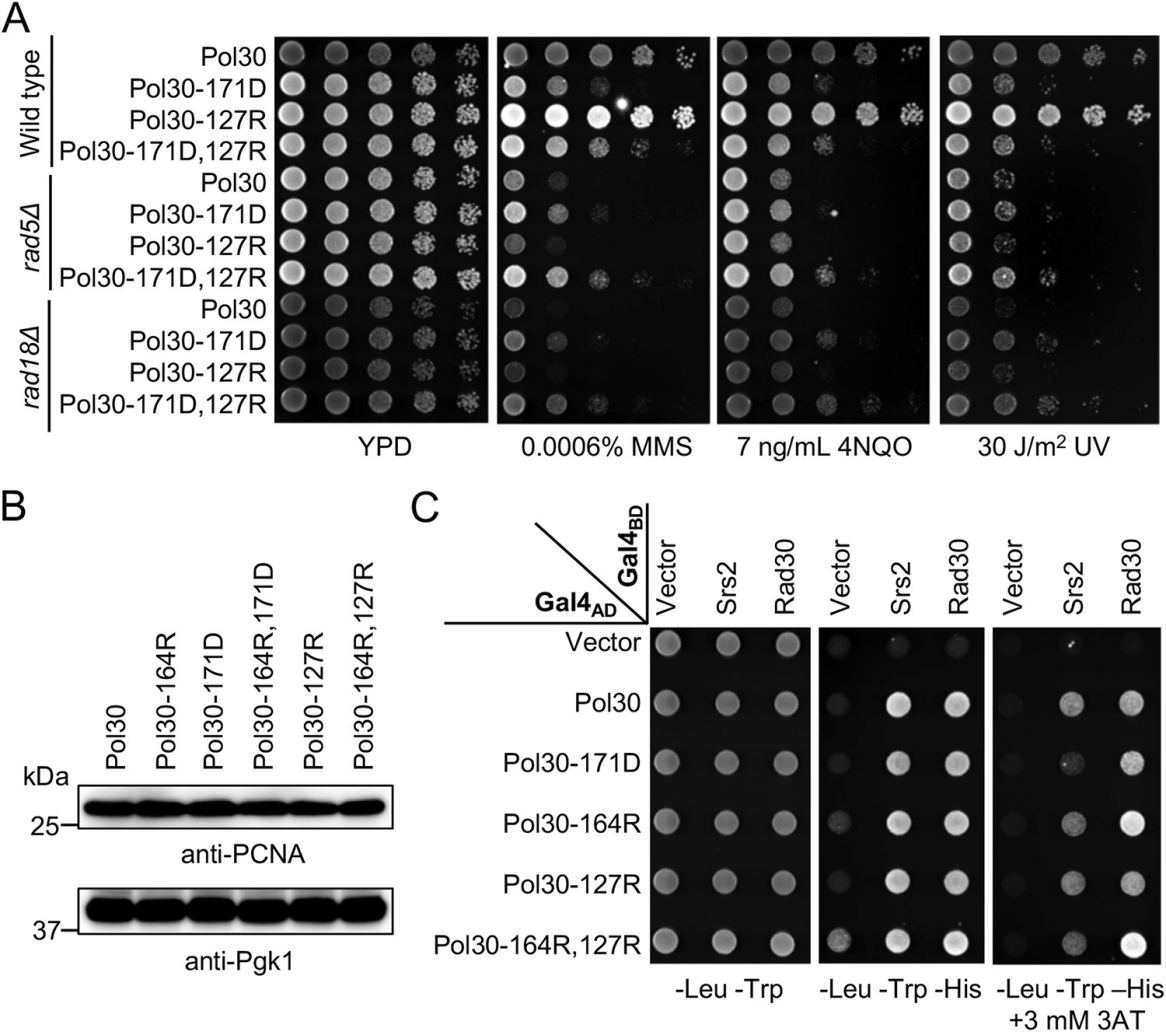
Functional characterization of the *pol30-K127R* mutation. (A) Genetic interaction between *pol30-K127R* and *pol30-A171D*. Experimental conditions are as described in Fig. 2. (B) Western blot analysis of cellular PCNA levels. Pol30 variants are as indicated on the top panel. Pgk1 was used as a loading control. (C) Physical interaction between Srs2 and Pol30 amino acid substitutions by a Y2H assay. Experimental conditions are as described in Fig. 3B, and the plates were incubated at 30°C for 3 days before photography.

Pol30-K127 can also be sumoylated (5), which regulates PIP-box proteins like EcoI for the sister chromatid cohesion (30). It was previously reported that *pol30-K127R* and *pol30-164R* mutations are synergistic in rescuing the *rad18* severe DNA-damage sensitivity (8, 9, 31), implying that sumoylation at the Pol30-K127 residue can backup Pol30-K164 sumoylation for the Srs2 recruitment. It raises a possibility that the Pol30-A171D substitution interferes with Pol30-K127 sumoylation, resulting in the observed mutant phenotypes. Indeed, under our experimental conditions, *pol30-K127R* itself does not display altered DNA-damage sensitivity or rescuing effects on *rad5* and *rad18* but causes synergistic phenotypes when combined with *pol30-K164R* (Fig. S2), reminiscent of the *pol30-A171D*,*K164R* double mutant (Fig. 2A,B). However, *pol30-A171D* and *pol30-K127R* are additive in DNA-damage response (Fig. 4A), indicating that they function in different pathways. The observed phenotypes were not due to altered cellular PCNA levels, as *pol30-A171D*, *pol30-K164R*, *pol30-K127R* and their corresponding double mutations do not affect protein stability (Fig. 4B). Furthermore, while Pol30-A171D fails to bind Srs2, Pol30-K127R and Pol30-K127R,K164R substitutions do not affect Srs2 interaction (Fig. 4C), further confirming that *pol30-A171D* rescues *rad5* and *rad18* independently of Pol30-K127 sumoylation.

10.1128/mbio.00315-23.4 FIG S2 Genetic interactions between *pol30-K164R* and *pol30-K127R* in rescuing severe DNA-damage sensitivities of *rad5* and *rad18* mutants by a serial dilution assay. Download FIG S2, DOCX file, 0.2 MB. Copyright © 2023 Fan et al. 2023 Fan et al. https://creativecommons.org/licenses/by/4.0/ This content is distributed under the terms of the Creative Commons Attribution 4.0 International license.

### Assessment of PCNA IDCL and C-terminal mutations.

**FIG 5:**
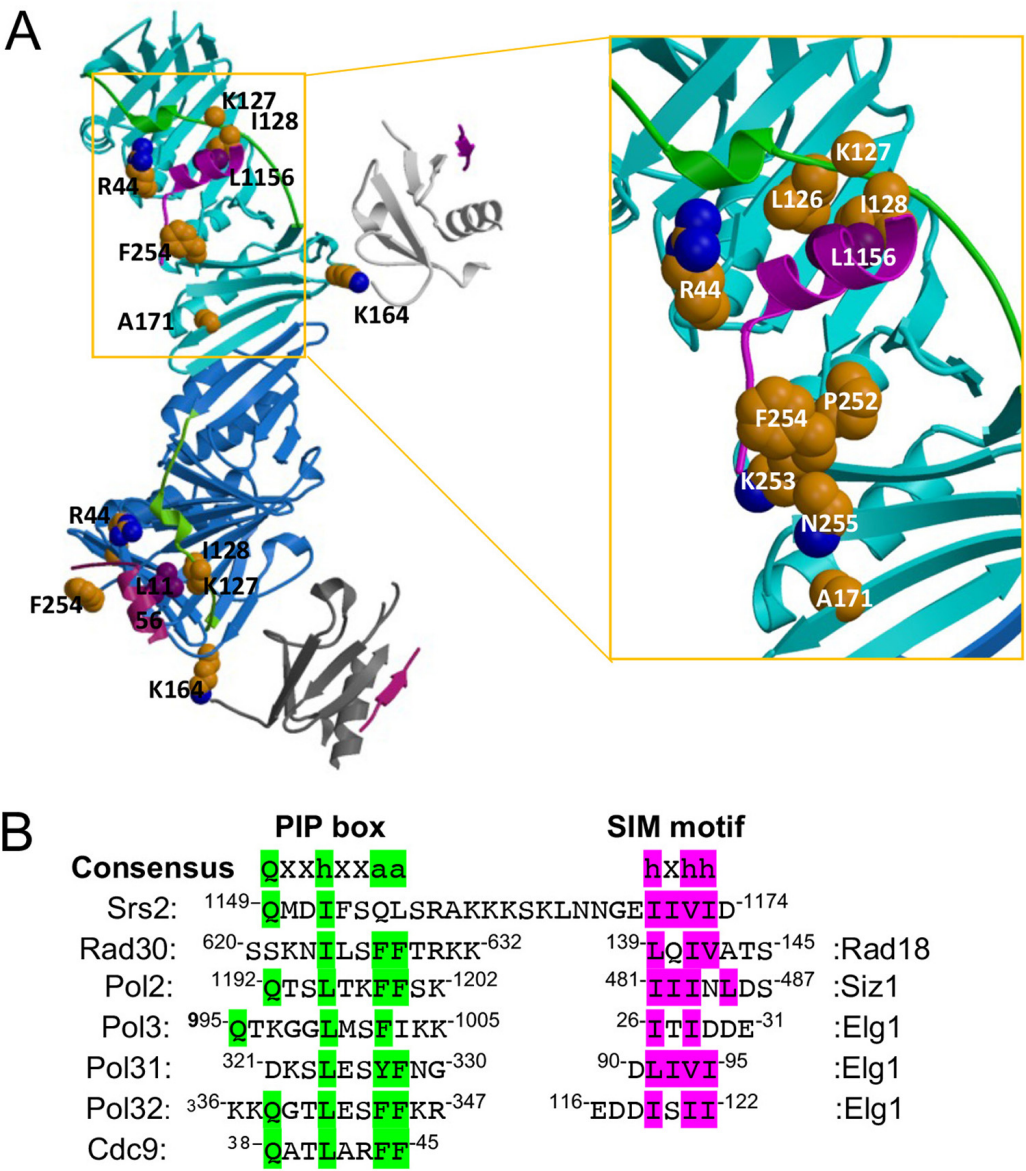
Protein structure and sequence analyses of the SUMO-PCNA-Srs2-CT complex. (A) The SUMO-PCNA-Srs2-CT complex structure from Protein Data Bank 3V62. Left panel: Two subunits of Pol30 (cyan and blue), SUMO (light and dark gray) and Srs2-CT (magenta, the linker between the PIP-like box and SIM is disordered) are shown. The two IDCL domains are highlighted in green. Right panel: an enlarged view from the boxed region on the left. Sidechains of discussed residues are shown in space-filling models with carbon atoms colored in gold. It is noted that in the deposited complex structure, PCNA-127 is a Gly instead of the anticipated Lys. (B) Amino acid sequences of Srs2 PIP box and SIM in comparison to PIP and SIM from other yeast proteins. Consensus sequences in PIP and SIM are highlighted in green and purple, respectively. “h” represents amino acids with moderately hydrophobic side chains like L, I, V and M; “a” represents amino acids with highly hydrophobic, aromatic side chains like F and Y; and “x” represents any residues.

To ask how the Pol30-A171D substitution affect its interaction with Srs2, we looked at the reported SUMO-PCNA-Srs2-CT complex structure (23). To our surprise, Pol30-A171 is not located in the PCNA-Srs2 interface (Fig. 5A). Srs2-CT has been reported to contain an “atypical PIP box” (23). Comparison of different sources of PIP-box sequences (Fig. 5B) revealed that among conserved residues, Srs2-CT contains an N-terminal “Qxxh” sequence, but not the C-terminal “FF” sequence. In contrast, some other proteins, including Rad30 contain the C-terminal “hxxFF” sequence but not the N-terminal “Q” residue. Since PIP box has been reported to bind two separate regions in PCNA, namely, IDCL (residues 121 to 132) and the C terminus (residues 251 to 258) (27), it is hypothesized that mutations in one of the two PCNA regions result in reduced PCNA-Srs2 interaction and the rescue of *rad5Δ/rad18*Δ mutant phenotypes. To test this hypothesis, two site-specific mutations were created: *pol30-2A* (L126A, I128A) was to affect the IDCL region and *pol30-4A* (252-255AAAA) was to affect the C-terminal region (Fig. 5A). In addition, *pol30-6A* (2A + 4A) was the combination of the above two mutations (Fig. 5A).

**FIG 6:**
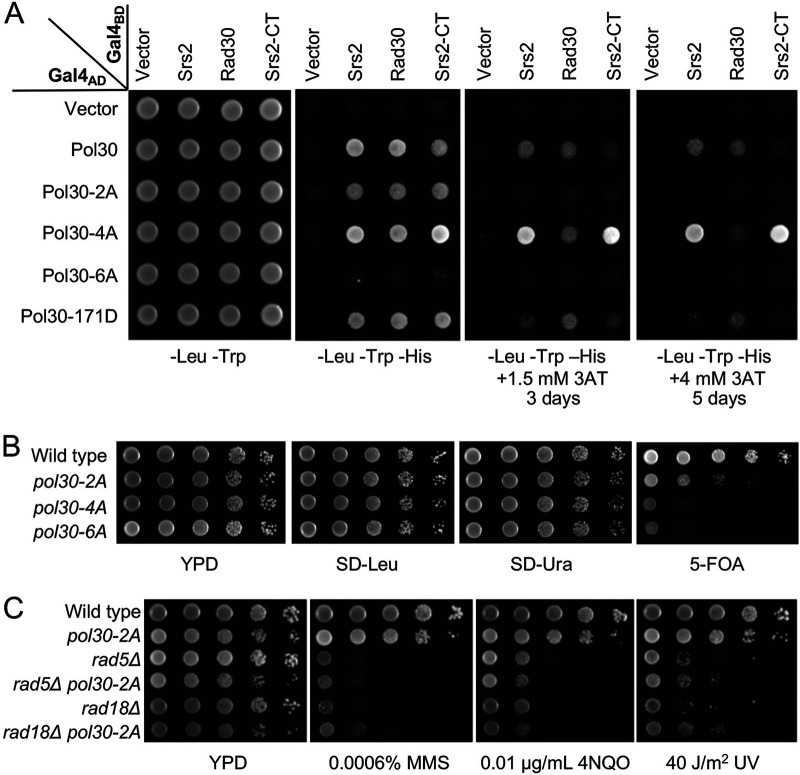
Phenotypes of Pol30 IDCL and C-terminal domain mutations. (A) Physical interaction between Pol30 mutations and Srs2 by a Y2H assay. Rad30 served as a reference. *SRS2*, *SRS2-CT* and *RAD30* were cloned into pGBT9 (Gal4_BD_) and Pol30 mutations that disrupt either IDCL (L126A, I128A = 2A) or the C terminus (252-255AAAA = 4A), or both (=6A) were cloned into pGAD424 (Gal4_AD_). Experimental conditions were as described in Fig. 3. (B) The viability assay for *pol30* mutants after plasmid shuffling. WXY939 cells transformed with YCpL-Pol30 derived plasmids were grown on YPD overnight to allow plasmid loss. Cells were then subjected to a serial dilution by spotting on SD-Leu to select those carrying YCpL-Pol30 based plasmid, on SD-Ura to select those carrying pBL211 (YCpU-POL30), and on 5-FOA to select cells that had lost the pBL211 plasmid. (C) Phenotypes of *pol30-2A* in rescuing *rad5Δ* or *rad18Δ* mutants from killing by DNA-damaging agents in a serial dilution assay. Experimental conditions were as described in Fig. 2. All plates were incubated at 30°C for 2 days before photography.

A Y2H assay (Fig. 6A) revealed that Pol30-2A reduced interaction with both Srs2 and Rad30; Pol30-4A affected Rad30 interaction but not Srs2 interaction, while Pol30-6A completely abolished interaction with both proteins.

To assess effects of the above mutations on the rescue of *rad5*Δ and *rad18*Δ severe DNA damage sensitivity, we attempted to shuffle plasmids with these *pol30* mutations. To our surprise, only *pol30-2A* cells were viable, albeit with reduced fitness, whereas *pol30-4A* and *pol30-6A* could not replace the plasmid carrying wild-type *POL30* with a *URA3* selectable marker (Fig. 6B). The above observations indicate that the PCNA C-terminal region plays an essential role, while its IDCL motif is important but dispensable for the cell viability.

The *pol30-2A* mutation alone caused moderate DNA-damage sensitivity and cannot rescue *rad5Δ* and *rad18Δ* cells from killing by DNA-damaging agents (Fig. 6C). Hence, a Pol30 mutation affecting interaction with both Srs2 and Rad30 cannot mimic the *pol30-A171D* phenotype.

### *pol30-I128A* can rescue *rad5Δ* and *rad18*Δ in a fashion reminiscent of *pol30-A171D*.

To search for a Pol30-A171D mimetic mutation and validate our hypothesis, a group of less destructive mutations were designed either within or around Pol30 IDCL and C-terminal regions based on the SUMO-PCNA-Srs2-CT complex structure (Fig. 5A). As Pol30-I128 buries Srs2-L1156, Pol30-I128A is expected to weaken IDCL binding to Srs2. Pol30-F254A should affect Srs2 interaction with the PCNA C terminus, as the Pol30-F254 sidechain buries Srs2-M1150, while equivalent residues in other PIP-containing proteins are hydrophilic (Fig. 5B). Therefore, Pol30-F254A would leave the pocket accessible to solvent (Fig. 5A). Srs2-F1153 is an aromatic residue with a Pi system unique among PIP-containing proteins, and Pol30-R44 may stabilize Srs2-F1153 with a cation/Pi interaction because of their large aromatic hydrophobic sidechain (32). In addition, Pol30-R44 is in a recently defined central loop (residues 41 to 44) (33). Hence, Pol30-R44A should disrupt this interface outside PCNA IDCL and C terminus (Fig. 5A).

**FIG 7:**
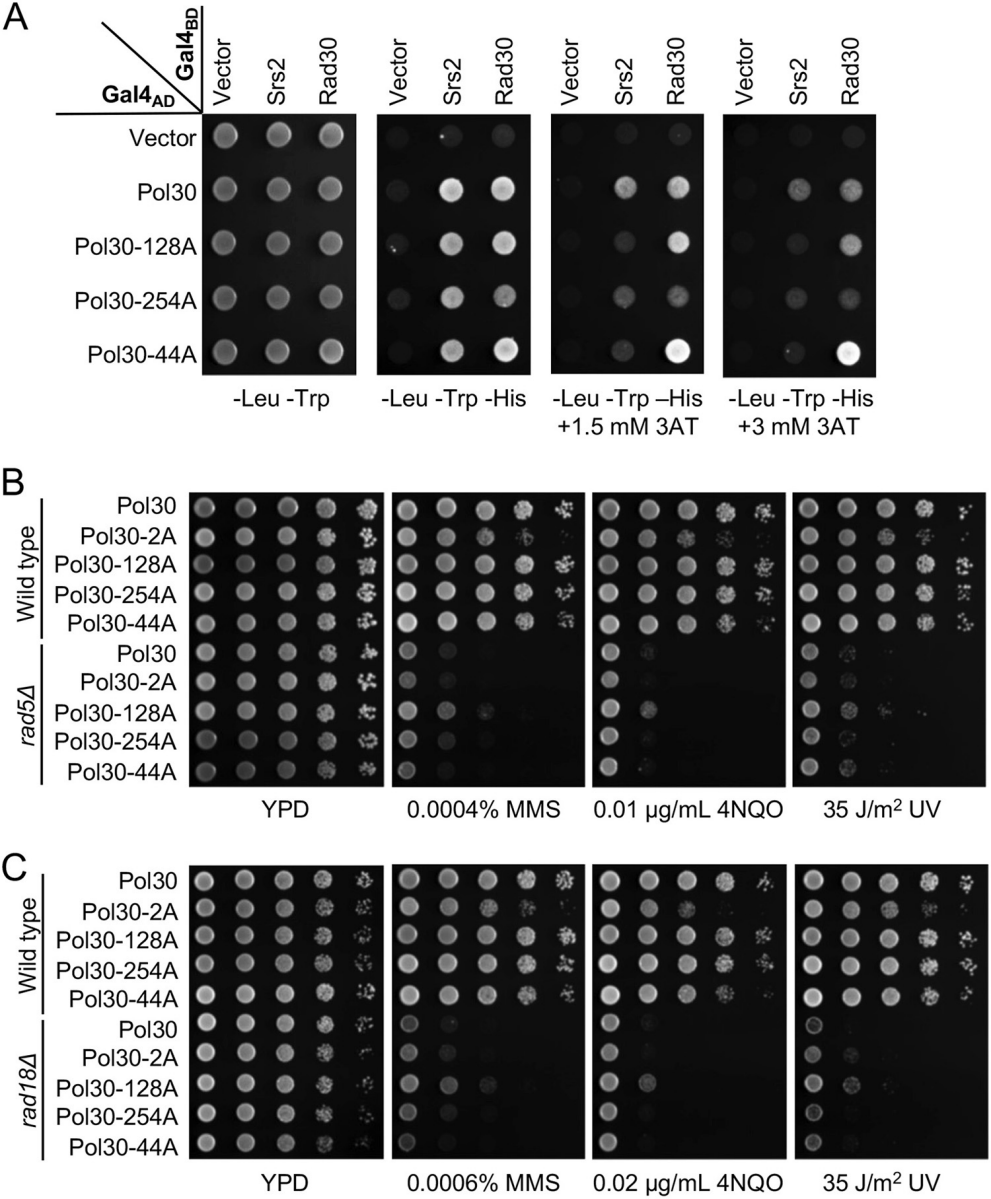
Phenotypes of selected *pol30* amino acid substitution mutants. (A) Physical interaction between *pol30* mutations and Srs2 or Rad30 by a Y2H assay. Experimental conditions were as described in Fig. 3. (B,C) Phenotypes of *pol30* mutants in rescuing *rad5*Δ (B) and *rad18*Δ (C) mutants from killing by DNA-damaging agents in a serial dilution assay. Experimental conditions were as described in Fig. 2. All plates were incubated at 30°C for 2 days before photography.

Like Pol30-A171D, Pol30-I128A reduced Srs2 but not Rad30 interaction in Y2H (Fig. 7A) and affinity pulldown (Fig. S3) assays. Meanwhile, Pol30-F254A reduced interaction with both Srs2 and Rad30, and Pol30-R44A decreased Srs2 interaction and enhanced Rad30 interaction (Fig. 7A). The above mutations were used to replace *POL30* in *rad5Δ* and *rad18Δ* cells, and the DNA damage sensitivity assay showed that *pol30-I128A* rescued both *rad5Δ* (Fig. 7B) and *rad18Δ* (Fig. 7C) cells by about 10-fold, which was comparable to *pol30-K164R* but less effective than *pol30-A171D*.

10.1128/mbio.00315-23.5 FIG S3 *In vitro* interaction between Srs2 C terminus (Srs2-CT), Rad30-PIP and Pol30 or its mutant derivatives by a GST pulldown assay. Download FIG S3, DOCX file, 0.2 MB. Copyright © 2023 Fan et al. 2023 Fan et al. https://creativecommons.org/licenses/by/4.0/ This content is distributed under the terms of the Creative Commons Attribution 4.0 International license.

**FIG 8:**
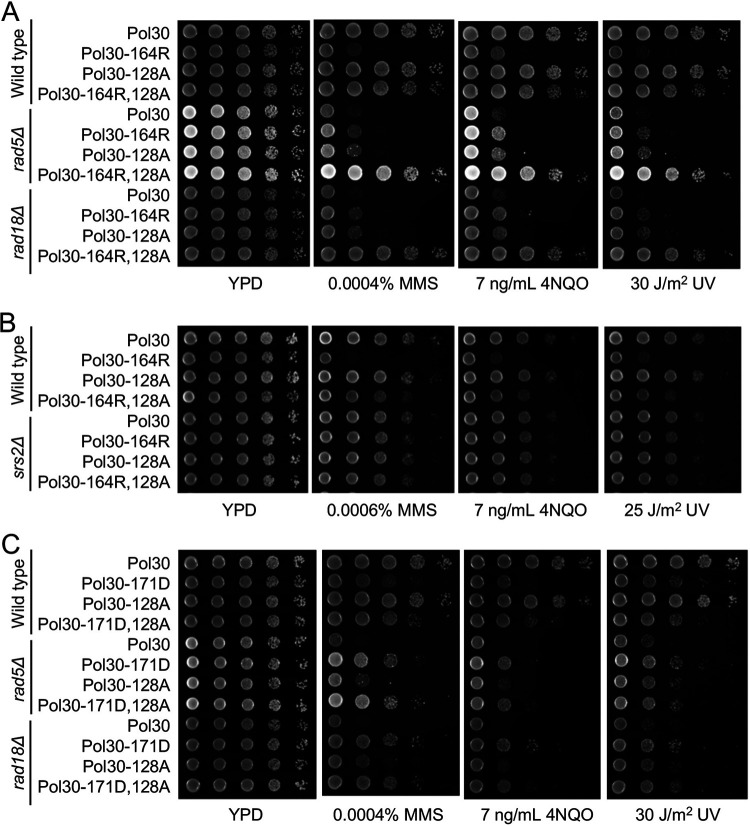
Genetic interactions between *pol30-I128A* and relevant mutations by a serial dilution assay. (A) Genetic interaction between *pol30-I128A* and *pol30-K164R* in the rescue of *rad5Δ* and *rad18*Δ mutants. (B) Genetic interaction between *pol30-I128A* and *pol30-K164R* in the *srs2* background. (C) Genetic interaction between *pol30-I128A* and *pol30-A171D* in the rescue of *rad5Δ* and *rad18*Δ mutants. Experimental conditions were as described in Fig. 2. All plates were incubated at 30°C for 2 days before photography.

To further address the genetic relationship between *pol30-I128A* and *pol30-K164R*, the corresponding double mutation was created, and the two single mutations were found to be strongly synergistic. While each single mutation rescued *rad5*Δ or *rad18*Δ DNA-damage sensitivity by about 10-fold, the double mutation rescued *rad5*Δ and *rad18*Δ by at least 1,000-fold under all experimental conditions, to the level indistinguishable from the double mutant alone (Fig. 8A), reminiscent of the *pol30-A171D*,*K164R* mutation. As expected, the *srs2* mutation is epistatic to *pol30-I128A* (Fig. 8B). Furthermore, the *pol30-A171D*,*I128A* double mutant behaved like the *pol30-A171D* and *pol30-I128A* single mutant (Fig. 8C), indicating that they rescued *rad5* and *rad18* severe sensitivity by the same mechanism. Like *pol30-A171D*, *pol30-I128A* also does not affect the PCNA-K164 sumoylation (Fig. S1, lanes 3 and 4). In summary, *pol30-I128A* mutant phenotypes are similar to *pol30-A171D*, allowing us to conclude that both *pol30-I128A* and *pol30-A171D* achieved *rad*5Δ and *rad18*Δ rescuing effects by specifically reducing interaction between PCNA and Srs2.

10.1128/mbio.00315-23.3 FIG S1 Western blot analysis to detect PCNA post-translational modifications. Download FIG S1, DOCX file, 0.08 MB. Copyright © 2023 Fan et al. 2023 Fan et al. https://creativecommons.org/licenses/by/4.0/ This content is distributed under the terms of the Creative Commons Attribution 4.0 International license.

## DISCUSSION

**FIG 9:**
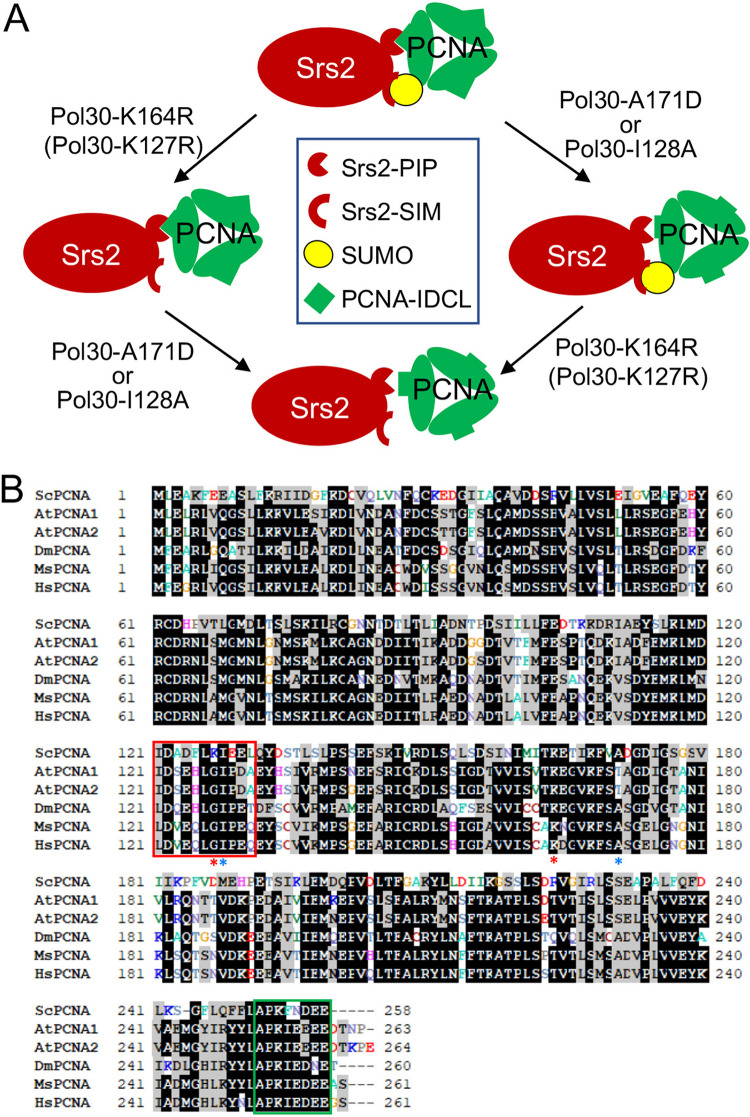
Critical PCNA residues in the recruitment of Srs2 and their conservation in other eukaryotes. (A) A working model on how Pol30-A171D/I128A and Pol30-K164R/K127R cooperatively disrupt physical interaction with Srs2. Srs2-CT contains a tandem receptor consisting of an atypical PIP box and a SIM domain that interact with PCNA IDCL and K164/K127-SUMO, respectively. While each single Pol30 mutation disrupt one such interaction, the corresponding double mutation fails to recruit Srs2, resulting in an equivalent *srs2* mutant phenotype in the rescuing of severe *rad5*Δ/*rad18*Δ DNA-damage sensitivity. (B) Multiple sequence alignment among PCNAs from selected eukaryotic model organisms and human. *Sc*, Saccharomyces cerevisiae; *At*, Arabidopsis thaliana; *Dm*, Drosophila melanogaster; *Mm*, Mus musculus; and *Hs*, Homo sapiens. Residues highlighted by black are identical sequences and those by gray are conserved sequences. PCNA IDCL and C-terminal regions are in red and green boxes, respectively. Pol30-A171 and -I128 residues are indicated by blue asterisks and Pol30-K164 and -K127 residues are indicated by red asterisks.

Previous reports have established that sumoylated PCNA facilitates the Srs2 recruitment, leading to the inhibition of unwanted HR (8, 9). This is achieved through coordinated interaction of Srs2-PIP and Srs2-SIM with PCNA and SUMO, respectively, forming a tandem receptor (23). Inactivation of *SRS2* can rescue the severe DNA damage sensitivity caused by malfunctional DDT in an HR dependent manner (18), a mechanism known as salvage HR (19, 20). Here, we report the isolation and systematic characterization of *pol30-A171D* that specifically reduces affinity for Srs2 to trigger salvage HR. First, an isolated *pol30-A171D* mutation can rescue not only *rad5*Δ, but also *rad18*Δ mutants from killing by DNA-damaging agents, indicating that this mutation induces salvage HR. Second, *pol30-A171D* does not affect PCNA sumoylationm and is synergistic with *pol30-K164R* in the rescuing, indicating that they act via distinct mechanisms. Third, since the *srs2* mutation is epistatic to *pol30-A171D* and *pol30-K164R*, both Pol30 substitutions are speculated to reduce Srs2 recruitment. Finally, *pol30-A171D* reduces physical interaction with Srs2; *pol30-K164R* is known to abolish PCNA sumoylation, while the double mutation losses both means to bind Srs2-CT (Fig. 9A), which explains why the two mutations confer synergistic effects in preventing Srs2 recruitment to the replication fork. Furthermore, this study rules out a possibility that *pol30-A171D* affects the Pol30-K127 sumoylation, leading to the synergistic interaction with *pol30-K164R*. First, *pol30-A171D* rescues the severe DNA-damage sensitivity of *rad5* and *rad18*, while *pol30-K127R* does not. Second, *pol30-A171D* and *pol30-K127R* are additive, indicating that they act in different pathways. Finally, Pol30-A171D reduces affinity for Srs2, while Pol30-K127R does not. The above observations support a notion that Pol30-K127 sumoylation can backup Pol30-K164 sumoylation in the recruitment of Srs2 (8, 9, 23) and hence both functions in the PCNA sumoylation branch (Fig. 9A).

One caveat for the above working model is that, based on the published SUMO-PCNA-Srs2-CT structure (23), Pol30-A171 is not located in the PCNA and Srs2-PIP box interface. To directly test our core hypothesis, a series of rationally designed *pol30* mutant derivatives were created and their ability to induce salvage HR as well as genetic interactions with *srs2* and *pol30-K164R* were examined, among which *pol30-I128A* functionally behaved like *pol30-A171D*. From the above complex structure, Pol30-I128 is located within the IDCL region in the PCNA-PIP interface, and the Pol30-I128A substitution specifically reduces Srs2 interaction but not Rad30 interaction, providing strong support to our working model (Fig. 9A). One can also see from the above structure that the sidechain of Pol30-A171 is buried in a pocket lined by four sidechains from Pol30-I158, -L151, -L154, -S155, and a β-sheet (Fig. 5A). Based on our collective observations and a speculation that Pol30-A171D also loosens PIP interaction, we predict that amino acid substitution from Pol30-A171 to a larger and hydrophilic Asp residue would destabilize the structure, which can be propagated to the Srs2-binding region(s).

Our Pol30 mutagenesis study reveals that PCNA binds Srs2-PIP and Rad30-PIP differently. First, Pol30-A171D selectively affects Srs2 but not Rad30 binding. Second, disruption of PCNA-IDCL by Pol30-2A reduces its interaction with both Srs2 and Rad30, while disruption of the PCNA C-terminal motif by Pol30-4A selectively interferes with Rad30 but not Srs2 interaction, indicating that the two PCNA regions preferentially bind different consensus sequences in the PIP box. Third, A single amino acid substitution in IDCL by Pol30-I128A has similar effects like Pol30-A171D, while Pol30-F254A in the C terminus only slightly reduces Rad30 binding. Finally, the Pol30-R44A substitution slightly reduces Srs2 interaction and enhances Rad30 binding, reminiscent of Pol30-K164R. The underlying mechanism appears to be that most PIP-boxes adopt a β-stranded structure at the N terminus followed by a 3_10_ helix at the C terminus, in which Cdc9-F45 is proximal to Pol30-L126 and I128 (27), whereas Srs2 residues ^1153^FSQL^1156^ adopt two turns of α-helix instead, in which Srs2-L1156 is proximal to Pol30-I128 and L131 (23). Our observation that Pol30-I128A reduces Srs2 interaction is consistent with a report that Srs2-L1156A also reduces its affinity for Pol30 (23). Apparently, Pol30-I128A would have only a moderate if any effect on the interaction with “FF” motif-containing PIP box proteins. In summary, if a Pol30 mutation only affects Srs2 interaction but not classical PIP box proteins (e.g., A171D and I128A), it could compromise Srs2 recruitment to the replication fork and activate the salvage HR. If a mutation affects both Rad30 and Srs2 (e.g., F254A) or only Rad30 but not Srs2 interaction (e.g., 2A), it cannot rescue the severe DNA-damage sensitivity of DDT mutants. Apparently, interaction with PIP-box proteins is an essential function for PCNA, as disruption of its C-terminal domain cannot maintain cell viability, a conclusion consistent with the structural and biochemical analyses (27). Fortunately, the Srs2-PIP is perhaps unique and hence targeted mutations in PCNA can be made to specifically disrupt its interaction without severe consequences on other PIP-box proteins.

Why have cells evolved such partial PIP boxes to be involved in the interaction with PCNA? One can imagine that proteins containing a full PIP box, including the Cdc9 DNA ligase, Polδ, and the Polε catalytic subunit Pol2, have relatively high affinity for, and constantly travel with, PCNA during replication. On the other hand, a partial PIP box may facilitate regulated association and dissociation from PCNA. For example, Srs2 is efficiently recruited by PCNA only when it is sumoylated (8, 9). Polη (Rad30) is recruited to the stalled replication fork when PCNA is monoubiquitinated at the same K164 residue (3) and can be dissociated when PCNA is no longer ubiquitinated (34). Interestingly, in addition to its genome replication role, Polδ is also involved in DDT (35 to 37), and some of its subunits contain partial PIP boxes (26). In response to DNA damage that blocks replication, a polymerase switch between Polδ (Pol3, Pol31 and Pol32) and Polζ_4_ (Rev3, Rev7, Pol31 and Pol32) takes place (38, 39). Whether this process involves PIP box regulation remains elusive.

Amino acid sequence alignment (Fig. 9B) reveals that PCNA IDCL and C-terminal regions are highly conserved throughout eukaryote organisms, from yeasts to human. The conserved residues include both Pol30-A171 and -I128 (Fig. 9B), suggesting that the regulatory mechanism as described in this study also applies to other eukaryotic organisms, although true Srs2 orthologs have not yet been identified in higher eukaryotes. Hence, this study sheds light on the investigation of DDT pathway regulation in higher eukaryotes, including plants and mammals.

## MATERIALS AND METHODS

### Yeast strains and cell culture.

S. cerevisiae cells were grown either in rich yeast-extract peptone dextrose (YPD) medium (1% yeast extract, 2% peptone, 2% glucose) or a synthetic dextrose (SD) medium (0.17% yeast nitrogen base without amino acids, 5% ammonium sulfate, 2% glucose supplemented with appropriate amino acids and bases). 2% agar was added when making plates. Plates containing 5-fluoroorotic acid (5-FOA) were made as previously described (29) to select for the *ura3* auxotroph.

Yeast strains used in this study are listed in Table S1. All strains used in this study were isogenic to HK578-10D except PJ69-4a (40). Gene deletion mutants were created by a one-step gene deletion method (41) using disruption cassettes as described (42), and the target gene deletion was confirmed by genomic PCR, followed by phenotypic analysis. Plasmids were transformed into yeast cells following a LiAc/single-strand carrier DNA/PEG method (43).

10.1128/mbio.00315-23.1 TABLE S1 *Saccharomyces* strains used in this study. Download Table S1, DOCX file, 0.02 MB. Copyright © 2023 Fan et al. 2023 Fan et al. https://creativecommons.org/licenses/by/4.0/ This content is distributed under the terms of the Creative Commons Attribution 4.0 International license.

### Plasmid construction and site-specific mutagenesis.

Plasmid YCpL-Pol30 was constructed by cloning the *POL30* open reading frame (ORF) along with its own promoter and terminator sequences into YCplac111 (YCp, *LEU2*) (44) as previously described (45). Site-specific *pol30* mutants were created in plasmid YCpL-Pol30 by a modified Quick Change method (46) using primers as shown in Table S2. The resulting plasmids were confirmed by sequencing the entire insert.

10.1128/mbio.00315-23.2 TABLE S2 Oligonucleotides used in this study. Download Table S2, DOCX file, 0.02 MB. Copyright © 2023 Fan et al. 2023 Fan et al. https://creativecommons.org/licenses/by/4.0/ This content is distributed under the terms of the Creative Commons Attribution 4.0 International license.

For the yeast two-hybrid (Y2H) assay, indicated yeast genes or fragments were amplified by PCR using primers containing restriction enzyme cleavage sites as listed in Table S2, cleaved by the corresponding restriction enzymes and cloned into either pGBT9 or pGAD424 (47) as Gal4_BD_ or Gal4_AD_ fusion, respectively. The Escherichia coli transformants were screened by colony PCR and the resulting plasmids were further confirmed by sequencing the entire insert.

To produce recombinant His_6_-tagged Pol30, plasmid pET-Pol30 was constructed by cloning the *POL30* ORF into BamHI-SacI sites of pET30a. To produce recombinant GST-tagged Srs2 C-terminal (Srs2-CT) domain, the DNA sequence encoding Srs2 residues 1107 to 1174 was amplified by primers Srs2-CT-BamHI-F and Srs2-CT-EcoRI-R (Table S2) and cloned into the BamHI-EcoRI sites of pGEX6. To produce recombinant GST-tagged Rad30-PIP, DNA sequences encoding Rad30 residues 515 to 632 was amplified by primers Rad30-BamHI-F and Rad30-EcoRI-R (Table S2) and cloned into the BamHI-EcoRI sites of pGEX6. All inserts in the cloned plasmids were confirmed by sequencing.

### Plasmid shuffling.

A plasmid shuffling method (29) was used to replace plasmid-borne wild-type *POL30* with *pol30* mutants. Briefly, yeast strain WXY939 and its isogenic mutants contain a chromosomal *pol30Δ::HIS3* allele and the cell viability was maintained by a plasmid pBL211 (YCp, *URA3*, *POL30*) (48), a gift from P. Burgers (University of Washington, St. Louis). To shuffle plasmids, WXY939 or its mutant cells were transformed with YCpL-Pol30 derived plasmids, resulting in transformants carrying two plasmids. The transformed cells were cultured in liquid YPD overnight, washed twice with sterile H_2_O, properly diluted and then 0.1 mL culture was spread on a 5-FOA plate. The plate was incubated at 30°C for 3 days and individual colonies were picked and streaked onto a fresh 5-FOA plate. Since the *URA3* gene product converts 5-FOA into a compound toxic to yeast cells (49), cells are able to grow only after they have lost the *URA3*-containing plasmid. Hence, the 5-FOA resistant cells were expected to only carry the YCpL-Pol30 plasmid or its mutant forms, whose phenotypes were then assessed. If the transformed cells do not form colonies on the 5-FOA plate, it is an indication that the *pol30* mutation carried by the YCpL-Pol30 plasmid cannot support the *POL30* essential function.

### Yeast cell survival assays.

The gradient plate and serial dilution assays were performed as previously described (50) to assess relative sensitivity of yeast cells to DNA-damaging agents. Briefly, for the gradient plate assay, overnight cultures were printed using a microscopic slide onto a set of two-layer YPD agar plates containing MMS concentration gradients. The plates were incubated for 2 to 3 days at 30°C before photography. For the serial dilution assay, overnight yeast cultures were used to make a set of 10-fold dilutions and then 4.5 μL samples were spotted on freshly made YPD agar plates containing different concentrations of MMS or 4NQO. UV treatment was achieved by exposing yeast cells in the spotted plate to UV irradiation in a UV cross-linker (Stratagene SS-UV1800) at given doses. All plates were incubated for 2 to 3 days at 30°C in the dark before photography. For both assays, only representative and informative plates were presented.

### Yeast two-hybrid assay.

Plasmids expressing Gal4_AD_ and Gal4_BD_ fusion proteins to be tested were cotransformed into yeast strain PJ69-4a. Transformants were allowed to grow at 30°C on an SD-Leu-Trp plate for 2 to 3 days, after which at least two groups of individual colonies were cultured and then spotted on an SD-Leu-Trp plate as control and on SD-Leu-Trp-His plates with or without certain concentrations of a histidine biosynthesis inhibitor 1, 2, 4-amino triazole (3AT) (51) to interrogate physical interaction between the two expressed gene products based on the relative growth on the selective plates. All plates were incubated for 2 to 6 days at 30°C before photography. Only representative and informative plates were shown.

### GST pulldown assay.

E. coli BL21 cells expressing His_6_-tagged Pol30 or GST-tagged Srs2-CT Rad30-PIP were induced by 0.1 mM IPTG at 16°C for 16 to 18 h. Cells were harvested by centrifugation, resuspended in corresponding His_6_ lysis buffer (50 mM Tris-HCl, pH 7.5, 150 mM NaCl, 30 mM imidazole) or GST lysis buffer (50 mM Tris-HCl, pH 8.0, 150 mM NaCl, 2 mM EDTA, 10 mM β-mercaptoethanol) and then homogenized in a cell disruptor (Constant Cell Disruption Systems, CF1) at 25 lb/in^2^ by two passes. Cells lysates were centrifuged at 16,000 *g* for 60 min. His_6_-Pol30 was affinity purified by Ni Sepharose (Cytiva, 17531801) and then eluted from the Ni Sepharose by 5-fold bed volume elution buffer (20 mM Tris-HCl, pH 7.5, 200 mM NaCl, 500 mM imidazole, 20% glycerol). GST-Srs2-CT and GST-Rad30-PIP were affinity purified by glutathione Sepharose (Cytiva, GE17-0756-01) in a GST stock buffer (50 mM Tris-HCl, pH 8.0, 150 mM NaCl, 2 mM EDTA, 10 mM β-mercaptoethanol, 20% glycerol). The above proteins were either freshly used or frozen in liquid nitrogen and stored at −80°C. Equal molars of GST-Srs2/Rad30 and His_6_-Pol30 were coincubated overnight at 4°C with gentle shaking. Anti-GST (Sigma, G7781-25UL, 1:1000), anti-His_6_ (New England Biolabs, 12698, 1:1000) primary antibodies and the anti-Rabbit (Bio-Rad, 1706515, 1: 3000) secondary antibody were used in immunoblotting.

### Western blot analyses.

Overnight cultured yeast cells were used to inoculate 50 mL fresh YPD at 1:20 dilution and the incubation continued at 30°C until OD_600nm_ =0.35 to 0.4. Cells were pelleted by centrifugation, washed with ddH_2_O and resuspended in 0.5 mL ddH_2_O, to which equal volume 0.2 M NaOH was added and incubated at 24°C for 15 min before adding 150 μL buffer containing 60 mM Tris-HCl, pH 6.8, 4% SDS, 0.01% bromophenol blue, 5% glycerol and 4% β-mercaptoethanol (52). The sample was boiled for 10 min, centrifuged at 15,000 rpm for 2 min and the supernatant was subjected to SDS-PAGE and Western blotting by using anti-PCNA (ab70472, Abcam, 1:1000) and anti-Pgk1 (a gift from Wei Li, Institute of Microbiology, Chinese Academic of Sciences) primary antibodies, and anti-mouse (Thermo Scientific 3140, 1:4000) and anti-rabbit (Bio-Rad, 1706515, 1: 5000) secondary antibodies, respectively.

### Protein structural and bioinformatics analyses.

The SUMO-PCNA-Srs2-CT complex structure (23) was obtained from the Protein Data Bank (PDB, https://www.rcsb.org/) entry 3v62 and displayed by using Molscript (53). The Cdc9-PCNA complex structure (27) was obtained from the PDB entry 2od8.

PCNA sequences were downloaded from NCBI (https://www.ncbi.nlm.nih.gov). Multiple alignments were performed and presented by BioEdit 7.2 downloaded from https://bioedit.software.informer.com/7.2/.
