# Supplementary material for: Genetic Dissection of Budding Yeast PCNA Mutations Responsible for the Regulated Recruitment of Srs2 Helicase
Source: mBio. 2023 Mar 2;14(2):e00315-23. doi: 10.1128/mbio.00315-23 (PMC10127746; doi:10.1128/mbio.00315-23)
Supplement: FIG S2 [file mbio.00315-23-s0004.docx]

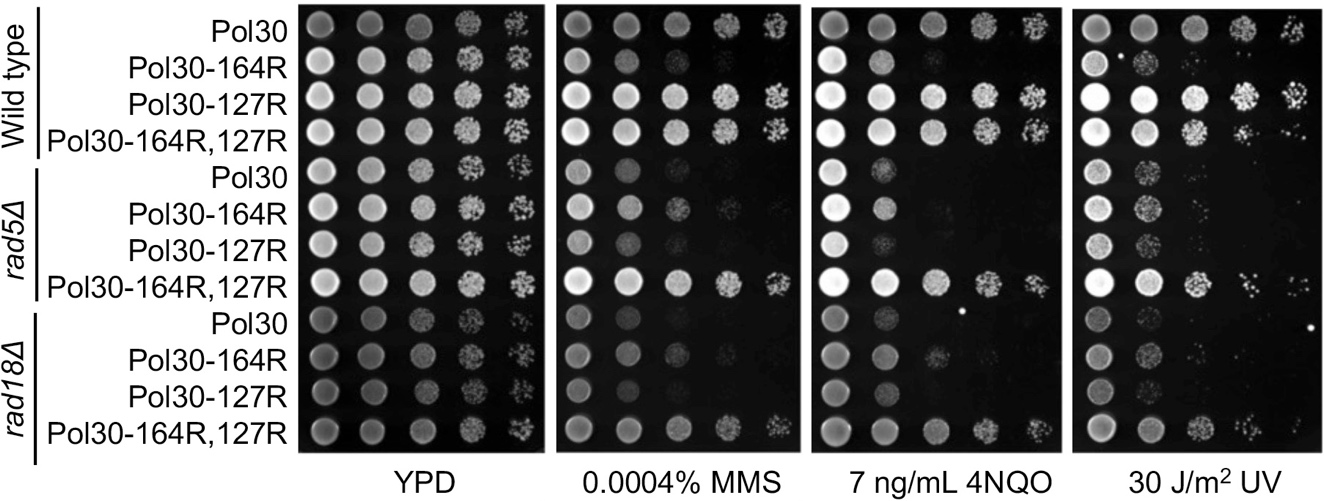


**FIG S2** Genetic interactions between *pol30-K164R* and *pol30-K127R* in rescuing severe DNA-damage sensitivities of *rad5* and *rad18* mutants by a serial dilution assay. Experimental conditions are as described in Fig. 2. All plates were incubated at 30 ℃ for 2 days before photography.
