# Supplementary material for: Genetic Dissection of Budding Yeast PCNA Mutations Responsible for the Regulated Recruitment of Srs2 Helicase
Source: mBio. 2023 Mar 2;14(2):e00315-23. doi: 10.1128/mbio.00315-23 (PMC10127746; doi:10.1128/mbio.00315-23)
Supplement: FIG S3 [file mbio.00315-23-s0005.docx]

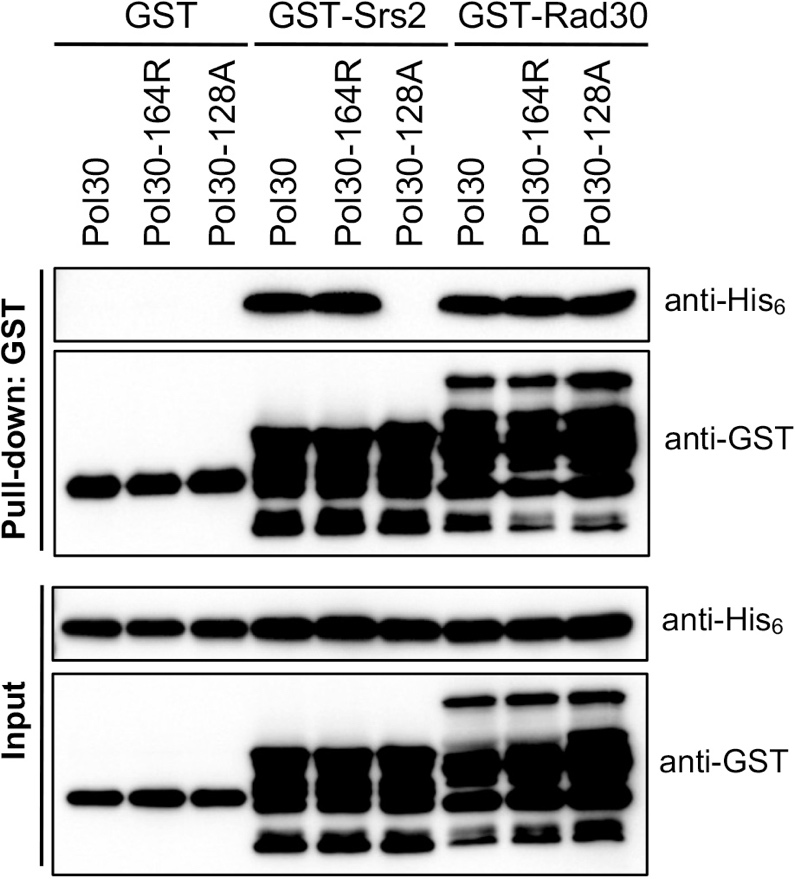


**FIG S3** *In vitro* interaction between Srs2 C-terminus (Srs2-CT), Rad30-PIP and Pol30 or its mutant derivatives by a GST pulldown assay. Proteins before and after the GST pulldown were subjected to western blotting by using antibodies against His_6_ and GST tags.
