# Supplementary material for: Genetic Dissection of Budding Yeast PCNA Mutations Responsible for the Regulated Recruitment of Srs2 Helicase
Source: mBio. 2023 Mar 2;14(2):e00315-23. doi: 10.1128/mbio.00315-23 (PMC10127746; doi:10.1128/mbio.00315-23)
Supplement: FIG S1 [file mbio.00315-23-s0003.docx]

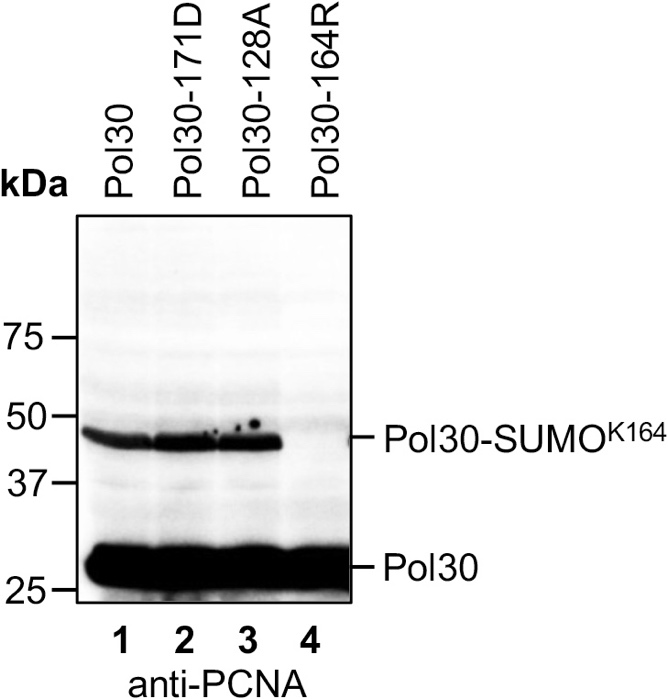


**FIG S1** Western blot analysis to detect PCNA post-translational modifications. WXY939 cells carrying the wild-type YCpL-Pol30 plasmid, and its mutant derivatives as indicated on the top panel were subjected to western blot analysis against anti-PCNA antibodies. PCNA and its sumoylated product are marked.
