# Supplementary material for: Genetic Dissection of Budding Yeast PCNA Mutations Responsible for the Regulated Recruitment of Srs2 Helicase
Source: mBio. 2023 Mar 2;14(2):e00315-23. doi: 10.1128/mbio.00315-23 (PMC10127746; doi:10.1128/mbio.00315-23)
Supplement: TABLE S1 [file mbio.00315-23-s0001.docx]

**SUPPLEMENTARY MATERIALS**

**TABLE S1.** *Saccharomyces* strains used in this study

| Strain | Genotype | Source |
| --- | --- | --- |
| HK578-10D | *MATα*, *leu2-3,112 trp1-1, can1-100, ura3-1, ade2-1, his3-11,15* | H. Klein |
| WXY939 | HK578-10D with *pol30Δ::HIS3*/pBL211 | Lab stock |
| WXY2972 | WXY939 with *rad5Δ::TRP1* | Lab stock |
| WXY978 | WXY939 with *rad18Δ::TRP1* | Lab stock |
| WXY999 | WXY939 with *srs2Δ::TRP1* | This study |
| PJ69-4a | *MATa*, *trp1-901 leu2-3,112 ura3-52, his3-200, gal4∆, gal80∆, GAL2-ADE2 LYS2::GAL1-HIS3, MET2::GAL7-lacZ* | P. James |
