# Supplementary material for: Genetic Dissection of Budding Yeast PCNA Mutations Responsible for the Regulated Recruitment of Srs2 Helicase
Source: mBio. 2023 Mar 2;14(2):e00315-23. doi: 10.1128/mbio.00315-23 (PMC10127746; doi:10.1128/mbio.00315-23)
Supplement: TABLE S2 [file mbio.00315-23-s0002.docx]

**TABLE S2.** Oligonucleotides used in this study

| Name | Sequence (5’-3’) |
| --- | --- |
| Pol30-A171D-F | GATCACCAAAGAAACAATAAAGTTTGTAGATGACGGTGATATCGG |
| Pol30-A171D-R | CTGAACCTGATCCGATATCACCGTCATCTACAAACTTTATTG |
| Pol30-K164R-F | GATTCTATTAATATCATGATCACCAGAGAAACAATAAAGTTTGTAGCTGACGGTGATATCG |
| Pol30-K164R-R | CTTTATTGTTTCTCTGGTGATCATGATATTAATAGAATCACTCAATTGGGACAAG |
| Pol30-164R171D-F | CATGATCACCAGAGAAACAATAAAGTTTGTAGATGACGGTGATATCGGATCAGGTTC |
| Pol30-164R171D-R | GATATCACCGTCATCTACAAACTTTATTGTTTCTCTGGTGATCATGATATTAATAGAATC |
| Pol30-K126,128AA-F | CGATGCTGATTTCGCGAAGGCGGAAGAATTACAGTACGACTCCACCC |
| Pol30-127R-F | GCTGATTTCTTAAGAATTGAAGAATTACAGTACGACTCC |
| Pol30-127R-R | CTGTAATTCTTCAATTCTTAAGAAATCAGCATCGATATCC |
| Pol30-K126,128AA-R | CTGTAATTCTTCCGCCTTCGCGAAATCAGCATCGATATCCATC |
| Pol30-251-255-AAA-F | CAGTTTTTCTTGGCTGCGGCGGCGGCTGACGAAGAATAA |
| Pol30-251-255-AAA-R | TTATTCTTCGTCAGCCGCCGCCGCAGCCAAGAAAAACTGTAGGAACCCACTC |
| Pol30-I128A-F | CGATGCTGATTTCTTAAAGGCGGAAGAATTACAGTACGACTCCACCC |
| Pol30-I128A-R | CTGTAATTCTTCCGCCTTTAAGAAATCAGCATCGATATCCATC |
| Pol30-F254A-F | CAGTTTTTCTTGGCTCCTAAAGCGAATGACGAAGAATAA |
| Pol30-F254A-R | TTATTCTTCGTCATTCGCTTTAGGAGCCAAGAAAAACTGTAGGAACCCACTC |
| Pol30-R44A-F | CATTGCACAAGCTGTCGATGACTCAGCGGTTCTATTGGTCTCC |
| Pol30-R44A-R | CCTATTTCCAAGGAGACCAATAGAACCGCTGAGTCATCGACAGC |
| Rad18∆-F | GAGCATCACAGCTACTAAGAAAAGGCCATTTTTACTACTCATGGAATTCCCGGGGATCC |
| Rad18∆-R | GCACAAGCTAACAAACAGGCCTGATTACATATACACACCAAGCTAGCTTGGCTGCAGGTCGA |
| Rad18∆-Check-F | GGTATAATCAATTGCAGAAGAGGCAGG |
| Rad18∆-Check-R | GTAGCAGGAACCGTAAACTACGCTGGCC |
| Srs2∆-F | CCAATTTGATCTTTCTTCTACCGGTACTTAGGGATAGCAAATGGAATTCCCGGGGATCC |
| Srs2∆-R | CCGCCTCCAATAGTTGACGTAGTCAGGCATGAAAGTGCTAAAGCTAGCTTGGCTGCAGGTCGAC |
| Srs2∆-Check-F | CCTGATACTACTGCTTAGGCTACCTTCGC |
| Srs2∆-Check-R | GGCATACTGCTCATTCATAGCTGTCATATCGG |
| Pol30-*Bam*HI-F | CGCGGATCCATGTTAGAAGCAAAATTTGAAGAAGCATCCC |
| Pol30-*Sac*I-R | CGAGCTCTTATTCTTCGTCATTAAATTTAGGAGCC |
| Pol30-*Pst*I-R | GCAGTTATTCTTCGTCATTAAATTTAGGAGCC |
| Srs2-CT-*Bam*HI-F | CGCCGCGGATCCATGCATAATCCGGATGACACTACAG |
| Srs2-CT-*Eco*RI-R | CCGGAATTCCTAATCGATGACTATGATTTCACCG |
| Srs2-CT-*Pst*I-R | GCATGCACTGCAGCTAATCGATGACTATGATTTCACCG |
| Srs2-*Bam*HI-F | CGCCGCGGATCCATGTCGTCGAACAATGATCTTTGG |
| Srs2-*Pst*I-R | GCATGCACTGCAGCTAATCGATGACTATGATTTCACCG |
| Rad30-*B*amHI-F | CGCCGC GGATCCATGGTTGTAGATATGTTTGGCAATCAGG |
| Rad30-*E*coRI-R | CCGGAATTCTCATTTTTTTCTTGTAAAAAATGATAAGATG |
| Siz1-*Bam*HI-F | CCGGAATTCATGATAAATTTAGAGGATTACTGGG |
| Siz1-*Pst*I-R | TGCACTGCAGTTAACCACTGTTGTATTTCTTTCCATAATCTTGCG |
